# Effect of V Content and Heat Input on HAZ Softening of Deep-Sea Pipeline Steel

**DOI:** 10.3390/ma15030794

**Published:** 2022-01-21

**Authors:** Ba Li, Qingyou Liu, Shujun Jia, Yi Ren, Ping Yang

**Affiliations:** 1 Engineering Steel Research Institute, Central Iron and Steel Research Institute, Beijing 100081, China; liuqingyou@cisri.com.cn (Q.L.); jiashujun@cisri.com.cn (S.J.); 15222951338@163.com (P.Y.); 2 State Key Laboratory of Metal Material for Marine Equipment and Application, Iron & Steel Research Institutes of Ansteel Group Corporation, Anshan 114009, China; angangry@126.com

**Keywords:** HAZ softening, weld thermal simulation, heat input, second-phase precipitation, effective grain size

## Abstract

In this paper, the welding thermal cycle process of deep-sea pipeline steel was investigated by welding thermal simulation. The microstructure evolution, crystallology and second-phase precipitation behavior of the soft zone of the heat-affected zone (HAZ) were characterized and analyzed by combining scanning electron microscopy, electron back-scattered diffraction, transmission electron microscopy and hardness testing. The results show that HAZ softening appeared in the fine-grained zone with a peak temperature of 900–1000 °C for deep-sea pipeline steel, the base metal microstructure of which was the polygonal ferrite and acicular ferrite. Using V microalloying and low welding heat input could effectively decrease the softening of the HAZ fine-grained region, which was achieved by reducing the effective grain size, increasing the proportion of the dislocation substructures, and precipitating the nanoscale second-phase particles.

## 1. Introduction

As is widely known, there are abundant reserves of marine oil and gas resources, approximately sixty percent of which discovered in recent years have been located in the ocean. Meanwhile, with the massive exploitation of terrestrial oil and gas resources and the increasing demand from humans, the exploitation of offshore oil and gas has attracted widespread attention [1,2]. Deep-sea pipeline steel is the main carrier of ocean oil and gas transportation, and is constantly subjected to various kinds of complex loads, including the loads generated by floating body movement, internal pressure generated by the passage of internal oil and gas, and ocean environmental loads such as wave loads and current loads. Therefore, the complex service environment puts forward more stringent performance requirements for deep-sea pipeline steel, including a high deformation strengthening index and uniform elongation, low yield strength ratio and good longitudinal mechanical properties [3,4,5]. It is usually necessary to adopt strain-based design for deep-sea pipeline steel, which usually has a dual-phase microstructure with a soft phase and hard phase. Thus, the high strain capacity of steel depends on the soft phase, and the strength depends on the hard phase for support, so as to ensure a good match between strength and plasticity [6,7].

However, the microstructure design of the dual-phase structure has caused the softening of the welding heat-affected zone (HAZ) of the deep-sea pipeline steel to become increasingly prominent [8,9,10]. The HAZ softening means that the hardness of a certain area in the welding HAZ is significantly lower than that of the base metal, so that the hardness distribution of the welded joint appears low in this area [8]. The appearance of the softening zone causes a serious mismatch of the performance between HAZ and the base metal, which becomes the weakest part of the entire welded joint, which seriously affects the service safety and service life of the welded structure [3,11,12]. 

Jia et al. [10] found that the softening occurred at the high-temperature region of HAZ for a ferrite + bainite dual-phase steel, while some researchers indicated that the softest region in HAZ was the fine-grained region of HAZ, especially for the fine-grained steel [8,9,11]. The formation of the softening zone is related to the phase transformation of the base metal microstructure under the welding thermal cycling, so the main influencing factors are the chemical composition of the base metal and the corresponding welding parameters [8,13,14,15]. Hamada et al. [14] pointed out that the carbon content had a closer relationship with HAZ softening than that of the P_cm_ (welding sensitivity coefficient) and C_eq_ (carbon equivalent) for an X80 UOE pipe, while Zhang et al. [16] indicated that Mo, Nb and V can improve the hardness of the softening zone to a certain extent for S690QL steels, while Ti has no obvious effect.

In the composition system of pipeline steel, V is not required to be added, in comparison with other elements such as Nb, Mo and Cr, etc. Among the three main microalloying elements, V has the best precipitation strengthening effect. Therefore, the precipitation of V-containing particles during thermal cycling will produce strong precipitation strengthening, which can effectively improve the hardness of microalloyed steel [17]. Moreover, it was reported that the VN and V (C, N) precipitations could provide nucleation sites for ferrite transformed along grain boundaries with a fine grain size during cooling. The ferrite could grow without coalescence resulting from the different orientations [9,18]. In addition, the thermodynamic calculation shows that the precipitation temperature of the second phase containing V is lower when V is added separately, while the composite addition of Nb and V can increase the precipitation temperature, which is beneficial to the secondary precipitation during the welding thermal cycle.

However, there are relatively few studies on the effect of V on the softening behavior of the welding HAZ of deep-sea pipeline steel with polygonal ferrite and an acicular ferrite mixed microstructure. 

## 2. Experimental Material and Procedures

In this work, the experimental steel was produced by industrially hot-rolled steel plates. The chemical composition of the experimental steel is listed in Table 1. Meanwhile, one deep-sea pipeline steel with a thickness of 31.8 mm, named 0# steel, was chosen to demonstrate the softening problem. The microstructure of the deep-sea pipeline steel consisted of polygonal ferrite and acicular ferrite. The chemical composition and mechanical property of 0# steel are displayed in Table 1 and Table 2, respectively. Based on the composite design of the deep-sea pipeline steel, the microalloying element V was added. The V content of the experimental design was 0.025, 0.050 and 0.070%, respectively. Then, 0.071% was the chemical composition after actual steelmaking. According to the principle of microalloying, the total addition of Nb, V and Ti usually does not exceed 0.1%. Therefore, we did not continue to add V. In addition, the microhardness of 0.05% V content was between 0.025 and 0.071. In order to clearly present the results of high and low V content, the results of 0.05% V were not added. The mechanical properties of experimental steel are also shown in Table 2, while the microhardness of the 1# and 2# base metal was 252 HV and 259 HV, respectively.

**Figure 1:**
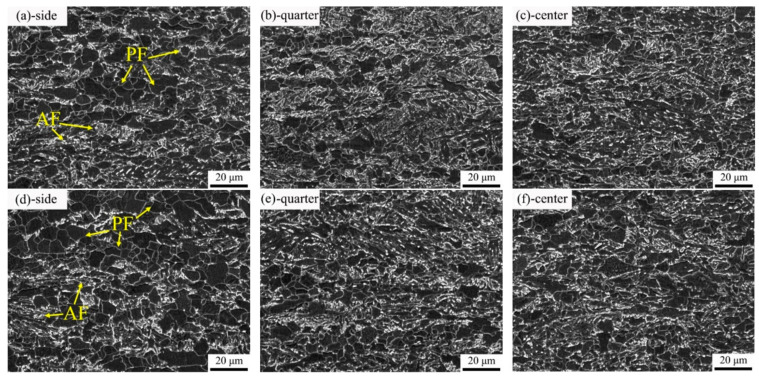
Base metal microstructure of experimental steel in thickness direction: (**a**–**c**) 1#-0.025V; (**d**–**f**) 2#-0.071V.

The base metal microstructure of the two experimental steels at different thickness is shown in Figure 1. Both of the two steels consisted of polygonal ferrite (PF) and acicular ferrite (AF). In addition, the content of PF from the side to the center of the two experimental plates was increased.

**Figure 2:**
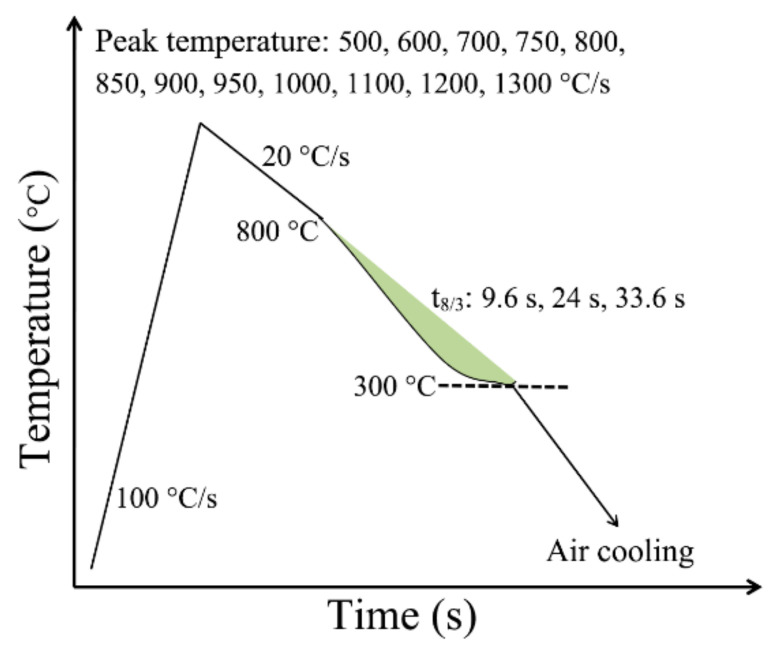
Schematic diagram of a hot welding simulation.

The schematic diagram of the thermal simulation process is shown in Figure 2. The thermal cycling process at different locations of HAZ was simulated by setting different peak temperatures through a Gleeble 1500 thermal simulation machine. The specimen was firstly reheated to different peak temperatures (500, 600, 700, 750, 800, 850, 900, 950, 1000, 1100, 1200 and 1300 °C, respectively) with a heating rate of 100 °C/s and then cooled to 800 °C with a cooling rate of 20 °C/s. The cooling time from 800 to 300 °C (t_8/3_) was 9.6, 24 and 33.6 s, respectively, corresponding to various welding heat inputs. Finally, the specimen was air-cooled from 300 °C to room temperature. The specimen size of the thermal simulation experiment was 11 mm × 11 mm × 60 mm, which was taken from the transverse side of the plate. It is worth noting that during the manufacture of the pipe, the steel plate obtained additional plastic deformations at macro-, meso-, and microlevels. These additional plastic deformations may cause local stress concentration, lead to local performance changes, and affect the analysis and judgment of experimental results. Therefore, the experimental specimen was directly cut down from the hot-rolled plates without deformation. In addition, different welding heat inputs of 10, 25, and 35 KJ/cm were used and the corresponding t_8/3_ times were 9.6, 24.95, and 33.6 s, respectively. The selection of welding heat input was combined with the actual welding conditions, and it was also an appropriate welding line energy range to avoid the local embrittlement of the coarse-grained zone. In particular, t_8/3_ was used instead of the commonly used t_8/5_ because the room-temperature microstructure of pipeline steels was usually a medium-temperature transformation microstructure.

The thermal simulation specimens were ground, polished, and etched in a 4% Nital solution. Then, the microstructure under different peak temperatures was represented by scanning electron microscopy (SEM, FEI Quanta 650FEG), transmission electron microscopy (TEM, HITACHI H-800) and electron back-scattered diffraction (EBSD, Oxford Nordlys F+). Meanwhile, the second-phase precipitation in the microstructure was observed and characterized by a high-resolution transmission electron microscope (HTEM, Tecnai G^2^F20). The TEM specimens were electropolished by the twin-jet electropolisher in an electrolyte with 6% perchloric acid and 94% methanol, while the specimens of the carbon extraction replicas for second-phase precipitate analysis were ground, polished, and etched in 4% Nital for 60–90 s. Then, a carbon film coating of about 20 nm was prepared on the surface by using an evaporator. The specimen surface with carbon film was carved into squares of about 2 × 2 mm^2^ in size by using a blade. The replicas were released in 4% Nital solution, cleaned in distilled water, and then placed on copper (Cu) grids. The EBSD specimens were electropolished by using the electrolyte consisting of 10% perchloric acid and 90% acetic acid. Both TEM and EBSD specimens were prepared at −30 °C. In addition, the microhardness of the experimental steel at each peak temperature was measured by microhardness tester (INSTRON TUKON 2100) with 8–12 measurements per point, and the load was 200 g.

## 3. Results and Discussion

### 3.1. Influence of V Content on HAZ Softening Zone of Submarine Pipeline Steel

**Figure 3:**
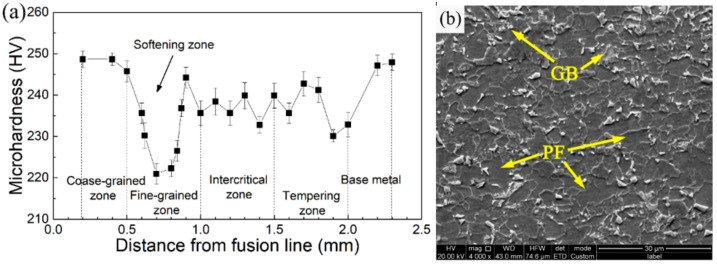
(**a**) Hardness distribution of HAZ in actual welded joint; (**b**) scanning electron micrographs (SEM) of microstructure in fine-grained HAZ.

Figure 3a shows the microhardness distribution of the HAZ of the actual welded joint of the 0# steel. It can be seen that there was an obvious softening zone at the position corresponding to the fine-grained HAZ, as marked by the black arrow. Then, as shown in Figure 3b, the microstructure of the fine-grained HAZ was transformed into a coarse microstructure mixed with granular bainite (GB) and PF, which led to a significant decrease in the strength and hardness, resulting in a softened zone.

**Figure 4:**
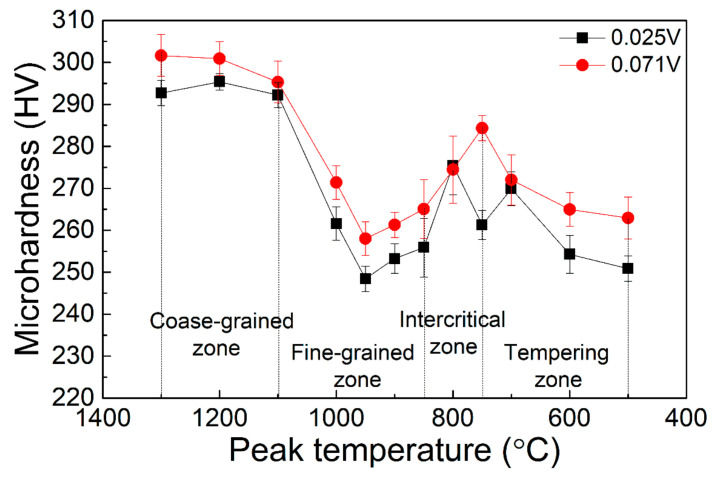
Relationship between peak temperature and microhardness under 10 KJ/cm heat input of two steels.

In order to alleviate the softening problem of the welding heat-affected zone, a V microalloying composition design was adopted. The microhardness distribution of the HAZ of the experimental steel after microalloying by V element is shown in Figure 4 under the thermal cycle of 10 KJ/cm heat input. It could be seen that the microhardness at all peak temperatures exceeded 245 HV, which was close to the hardness of the base metal. Therefore, the depth of the softening zone in the fine-grained HAZ of the V-containing experimental steel was obviously shallower. Moreover, with the increase in the V content from 0.025 to 0.071, the degree of shallowness of the softening zone became more obvious.

**Figure 5:**
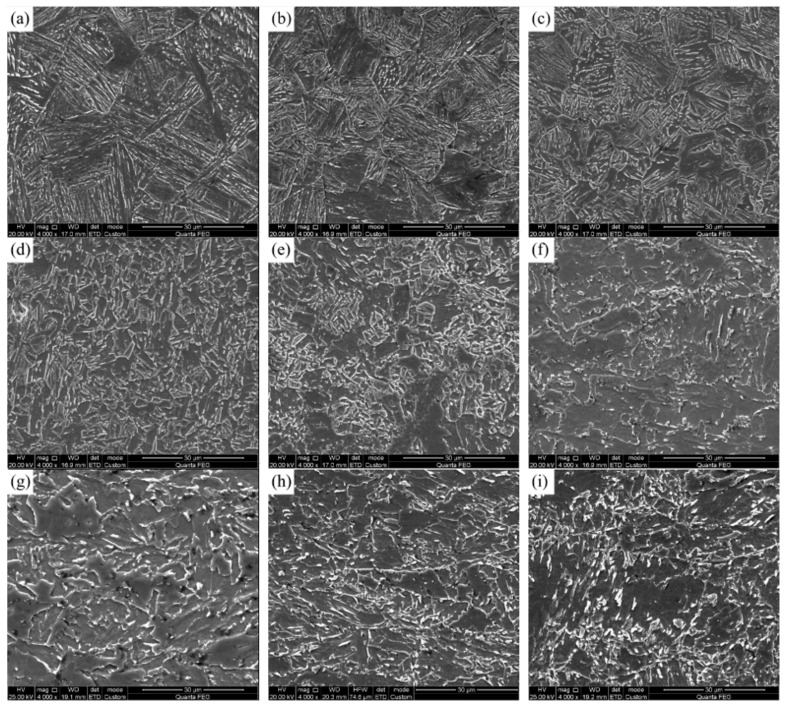
SEM of different peak temperatures for 1# steel with 0.025% V under 10 KJ/cm heat input: (**a**) 1300 °C; (**b**) 1200 °C; (**c**) 1100 °C; (**d**) 1000 °C; (**e**) 900 °C; (**f**) 800 °C; (**g**) 700 °C; (**h**) 600 °C; (**i**) 500 °C.

Figure 5 shows the SEM pictures of the microstructure of 1# steel with 0.025% V content at different peak temperatures under 10 KJ/cm heat input. The peak temperature of 1300 °C simulated the coarse-grained zone in the HAZ near the fusion line. As shown in Figure 5a, the microstructure was dominated by bainite ferrite (BF) and contained a small amount of GB. The microhardness of BF was much higher than both GB and AF. The microstructure in this temperature region had undergone complete higher austenitizing. Then, the austenite grains were coarsened, and the austenite stability was also high. Therefore, the final microstructure after rapid cooling was dominated by BF with relatively coarse grain sizes. Meanwhile, the parent austenite grain boundaries were clearly visible. As the peak temperature of the coarse-grained zone decreased (1200–1100 °C), that is, the austenitizing temperature decreased, the GB content in microstructure increased after cooling, but it was still dominated by BF. However, the degree of the microstructure coarsening was decreased, as shown in Figure 5b,c. When the peak temperature was reduced to 1000–900 °C, corresponding to fine-grained HAZ, as shown in Figure 5d,e, the austenitizing temperature was lower, and the austenite stability was also low. As a result, the microstructure did not coarsen significantly, resulting in a PF, AF and GB mixed microstructure after rapid cooling. Compared with the fine-grained HAZ in the actual welded joint, the increase in the proportion of GB and AF in the microstructure of 1#-0.025 steel could improve the microhardness. As the peak temperature of the fine-grained zone decreased, the structure was not adequately austenitized, resulting in an uneven local distribution of microstructure, as shown in Figure 5e. As the peak temperature decreased to the intercritical zone of 800 °C (Figure 5f), the PF in the parent microstructure was austenitized first, and the rest of AF recovered to a certain extent. After cooling, the austenitized part transformed into GB, and the final microhardness in this zone was still high. Moreover, the peak temperature of 700–500 °C (Figure 5g,i) was the tempering zone in which the microstructure did not austenitize. Therefore, the hardness was not significantly reduced in the tempering zone due to a certain degree of microstructure recovery occurring. In summary, the addition of 0.025% V to the composition of deep-sea pipeline steel increased the bainite-type structure (such as AF and GB) in the fine-grained HAZ, thereby increasing the hardness and making the softening pit shallow.

**Figure 6:**
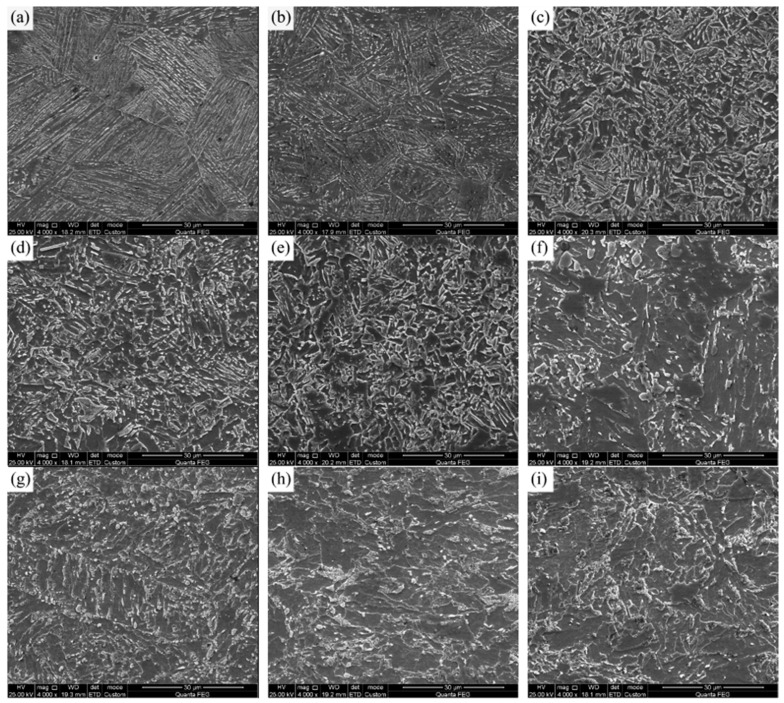
SEM of different peak temperatures for 2#-0.071V steel under 10 KJ/cm heat input: (**a**) 1300 °C; (**b**) 1200 °C; (**c**) 1100 °C; (**d**) 1000 °C; (**e**) 900 °C; (**f**) 800 °C; (**g**) 700 °C; (**h**) 600 °C; (**i**) 500 °C.

**Figure 7:**
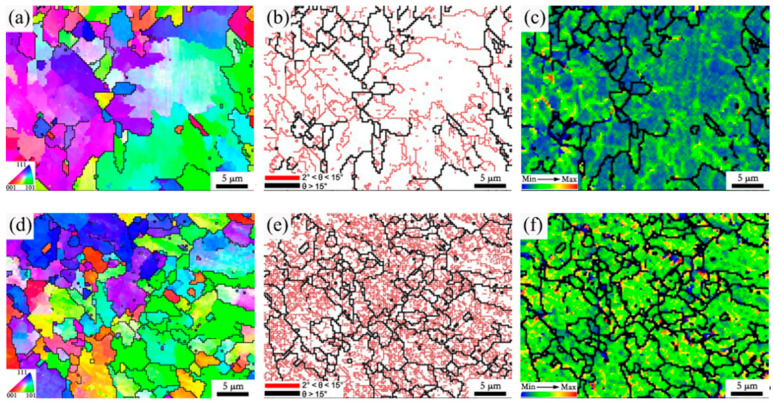
EBSD maps of microstructure in fine-grained HAZ (950 °C) for experimental steel with different V contents under 10 KJ/cm heat input: (**a**) 1#—inverse pole figure; (**b**) 1#—grain boundary map; (**c**) 1#—local misorientation distribution map; (**d**) 2#—inverse pole figure; (**e**) 2#—grain boundary map; (**f**) 2#—local misorientation distribution map.

Figure 6 shows the SEM pictures of the microstructure of 2# steel with 0.071% V content at different peak temperatures under 10 KJ/cm heat input. Compared with the microstructure in Figure 5, it can be seen that the change in the microstructure of the coarse-grained zone with the peak temperature of 1300–1100 °C and the intercritical zone and tempering zone with the peak temperature of 800–500 °C was similar to that of the 1# experimental steel. However, the fine-grained zone microstructure at 900 °C of 2# steel was fine and uniform, as shown in Figure 6e. In order to further explore the reason why the softening pits of the fine-grained HAZ became shallower due to the increase in V content, the microstructure at the lowest position in the fine-grained zone of 950 °C was characterized by EBSD, as shown in Figure 7.

**Figure 8:**
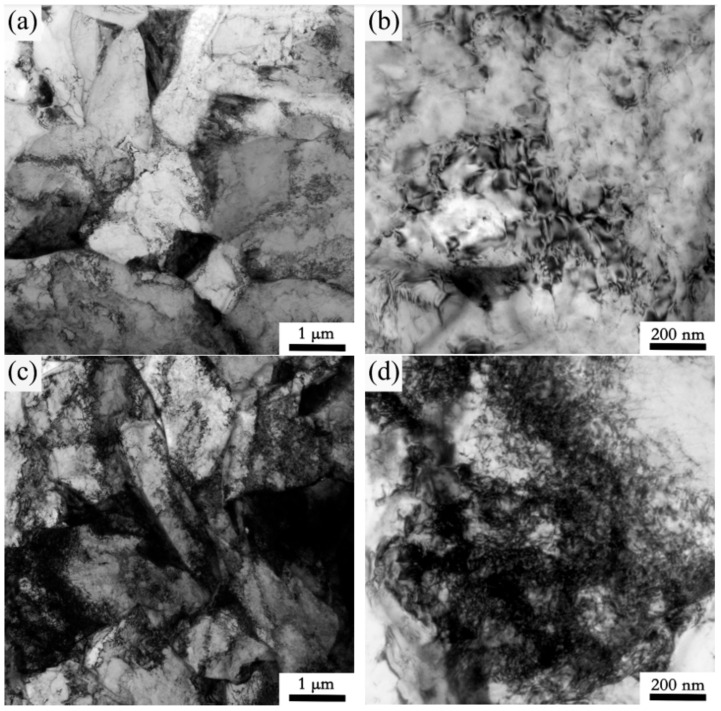
TEM maps of microstructure in fine-grained HAZ (950 °C) for experimental steel with different V contents under 10 KJ/cm heat input: (**a**) subgrain structures in 1# steel (**b**) dislocation structures in 1# steel; (**c**) subgrain structures in 2# steel (**d**) dislocation structures in 2# steel.

Figure 7a,d show the inverse pole figures of the two experimental steels. The large ferrite in the fine-grained zone of 1# steel and the fine ferrite in 2# steel can be clearly seen. It can be seen from the corresponding grain boundary maps that the proportion of low-angle grain boundaries (with a critical angle between 2° and 15°) in the microstructure of 2# steel (Figure 7b) was significantly higher than that of 1# steel (Figure 7e). Moreover, Figure 7c,f shows the local misorientation distribution maps of the microstructure in the fine-grained HAZ of two experimental steels under 10 KJ/cm heat input. This function of EBSD is to characterize the change in the content and distribution of a misorientation of less than 5° inside the grain through a change to a rainbow color, which can reflect the degree of local stress concentration in the microstructure [19]. In addition, it can also be used to indirectly characterize the change in dislocation density [20]. According to the figure legend, the color changed from blue to green to orange, indicating that the degree of stress concentration in the microstructure was increasing, that is, the dislocation density was increasing. Compared with 1# steel, the whole microstructure of 2# steel had a high dislocation density, resulting in a high microhardness. Therefore, as the V content in the base metal increased, the significant increase in dislocation density was one of the reasons for the reduced softening of the fine-grained HAZ. Moreover, the substructure and dislocation configuration in the fine-grained HAZ of the two experimental steels can be directly observed through TEM. As can be clearly seen in Figure 8, compared with 1# steel, 2# steel had rich substructures of small size (Figure 8a,c), and a high density of dislocations in the microstructure (Figure 8b,d), which was consistent with the observation of EBSD.

**Figure 9:**
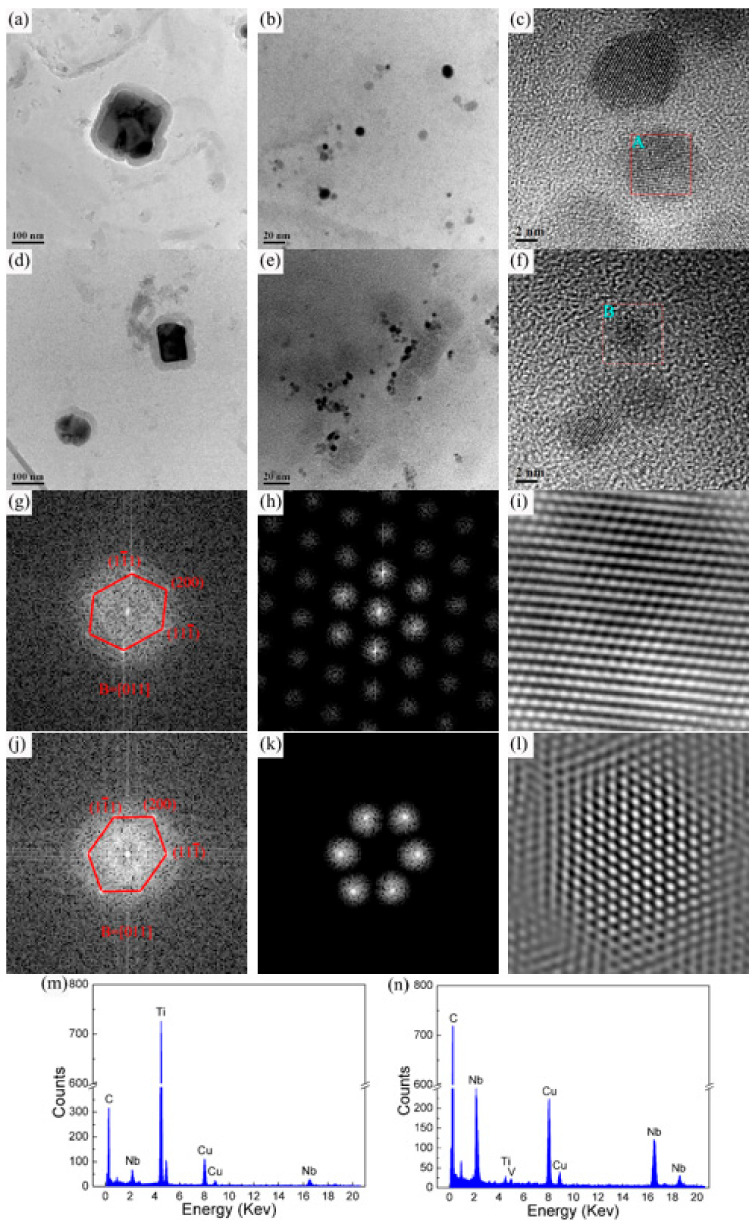
Precipitation and energy spectrum of experimental steel at fine-grained HAZ (950 °C) with different V contents under 10 KJ/cm heat input: (**a**–**c**) 1# steel with 0.025% V; (**d**–**f**) 2# steel with 0.071% V; (**g**–**i**) high-resolution photos of particle A; (**j**–**l**) high-resolution photos of particle B; (**m**,**n**) energy spectra of two types of precipitation.

Figure 9 shows the second-phase precipitation of the two experimental steels in the fine-grained zone (950 °C, 10 KJ/cm). There were two types of precipitated particles in the microstructure. One type was a square second phase larger than about 100 nm (Figure 9a,d). The energy spectrum shows that this large second phase was (Nb, Ti) C particles enriched with Ti, as shown in Figure 9m, which were coarsened from the undissolved second phase in the base material during the thermal cycle. This type of second phase particles was very small in number and had little effect on the overall strength and hardness of the microstructure. Another type of precipitate in microstructure was a spherical second phase smaller than about 10 nm, as shown in Figure 9b,e. This nanoscale second-phase particle was a (Nb, Ti, V) C composite precipitation phase with Nb enriched through the energy spectrum results (Figure 9n). Figure 9g–i,j–l present their corresponding high-resolution photos, showing that there was no essential difference between the precipitate in the lattice distribution of the two steels. As we all know, the nano-precipitated phase with a size of less than 10 nm is beneficial to improve the strength and hardness, and has a significant second phase strengthening function [20]. Therefore, the second phase strengthening of the nano-precipitated phase was an important reason for reducing the softening of the fine-grained HAZ. Further comparing Figure 9b,c,e,f, it can be seen that with the increase in V content, the number of nanoscale second-phase particles increased, the size decreased, and the second phase strengthening effect was enhanced, thereby further reducing the softening of the fine-grained HAZ. Related studies have pointed out that when a large number of microalloyed carbonitrides precipitate in HAZ, it will consume the carbon in the austenite, which will reduce the hardness of the M/A constituent and cause softening [18]. However, from the analysis of the microstructure in the fine-grained zone above, the microstructure was significantly refined after V was added to the base material, and the M/A constituent was also refined, so the hardness had little effect.

### 3.2. Influence of Heat Input on HAZ Softening Zone of Deep-Sea Pipeline Steel

**Figure 10:**
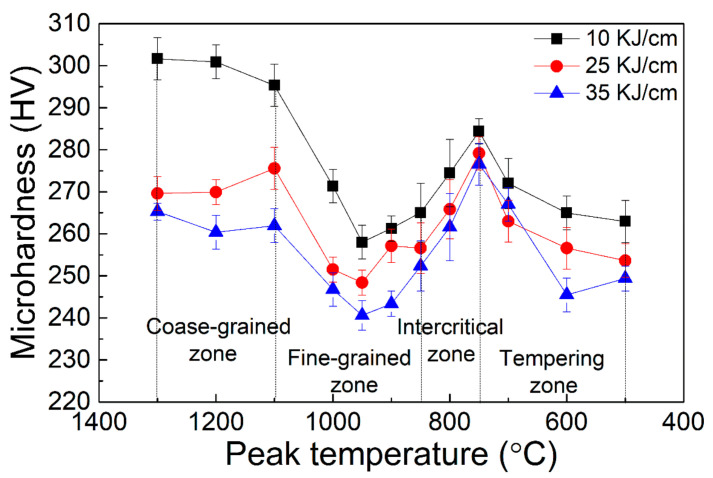
HAZ microhardness of 2# steel with 0.071% V under different heat inputs.

Figure 10 shows the microhardness of the heat-affected zone (HAZ) of 2# steel with 0.071% V under different heat inputs. It can be seen that reducing the welding heat input could make the softening pits in the fine-grained zone significantly shallower.

**Figure 11:**
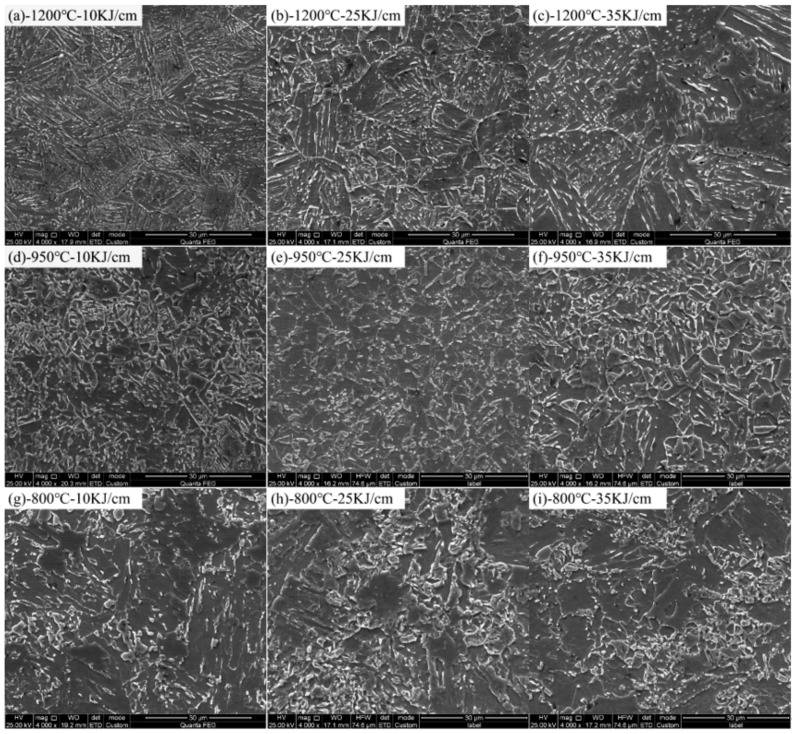
SEM of 2# steel with 0.071% V: (**a**–**c**) peak temperature of 1200 °C under the heat input of 10 KJ/cm, 25 KJ/cm and 35 KJ/cm, respectively; (**d**–**f**) peak temperature of 950 °C under heat input of 10 KJ/cm, 25 KJ/cm and 35 KJ/cm, respectively; (**g**–**i**) peak temperature of 800 °C under heat input of 10 KJ/cm, 25 KJ/cm and 35 KJ/cm, respectively.

Figure 11 shows the 2# steel with 0.071% V microstructure at different peak temperatures under different heat input conditions. As the heat input increased, the cooling rate in HAZ decreased, while the microhardness of the BF microstructure was higher than that of GB and AF. Therefore, in the coarse-grained zone of 1200 °C (Figure 11a–c), with the increase in heat input, both the proportion of GB and the grain size of microstructure increased, which made the hardness significantly decrease, while in the fine-grained zone of 950 °C, GB content decreased and PF content increased with the increase in heat input, as shown in Figure 11d–f. As a result, the degree of softening of the fine-grained HAZ increased with the increase in heat input. When the peak temperature dropped to the intercritical zone of 800 °C (Figure 11g–i), at the low heat input of 10 KJ/cm, some of the austenitized microstructures transformed into granular bainite after rapid cooling, while the untransformed microstructures recovered to a certain extent. Therefore, microhardness increased slightly, as shown in Figure 10. However, as the heat input increased from 25 to 35 KJ/cm, part of the austenitized microstructures gradually transformed into ferrite, resulting in a decrease in microhardness to some extent.

**Figure 12:**
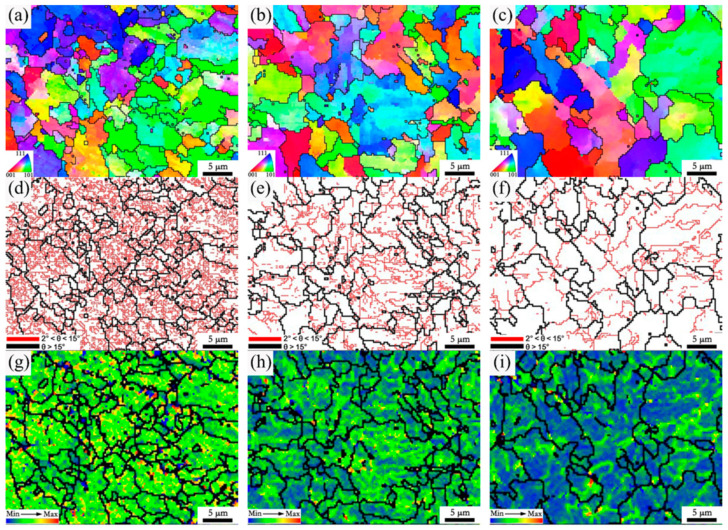
IPF of microstructure in fine-grained HAZ of 950 °C for 2# steel with 0.071% V under different heat inputs: (**a**) 10 KJ/cm; (**b**) 25 KJ/cm; (**c**) 35 KJ/cm. Grain boundary maps: (**d**) 10 KJ/cm; (**e**) 25 KJ/cm; (**f**) 35 KJ/cm. Local misorientation distribution maps: (**g**) 10 KJ/cm; (**h**) 25 KJ/cm; (**i**) 35 KJ/cm.

Figure 12 presents the EBSD maps of the fine-grained HAZ of 2# steel with 0.071% V under different heat inputs. It can be seen that with increases in heat input, both the proportion and grain size of ferrite increased, resulting in the effective grain size of the microstructure increasing in the fine-grained HAZ (Figure 12a–c). Meanwhile, the proportion of low-angle grain boundaries in the microstructure was greatly reduced with the increase in heat input, as shown in Figure 12d–f. The local misorientation distribution maps corresponding to the different heat inputs show that the dislocation density decreased significantly as the heat input increases, as shown in Figure 12g–i. Guo et al. [21] performed EBSD characterization on the HAZ microstructure of the ultra-low carbon Nb-containing acicular ferrite steel, then pointed out that the low-angle grain boundary with a misorientation of less than 3° reflected the aggregation of high-density dislocations. Therefore, both the decrease in the proportion of low-angle grain boundaries and the decrease in dislocation density could reduce the microhardness, resulting in the occurrence of a softening zone under high heat input. In addition, the effective grain size increased under high heat input, which was also harmful in reducing the softening phenomenon.

**Figure 13:**
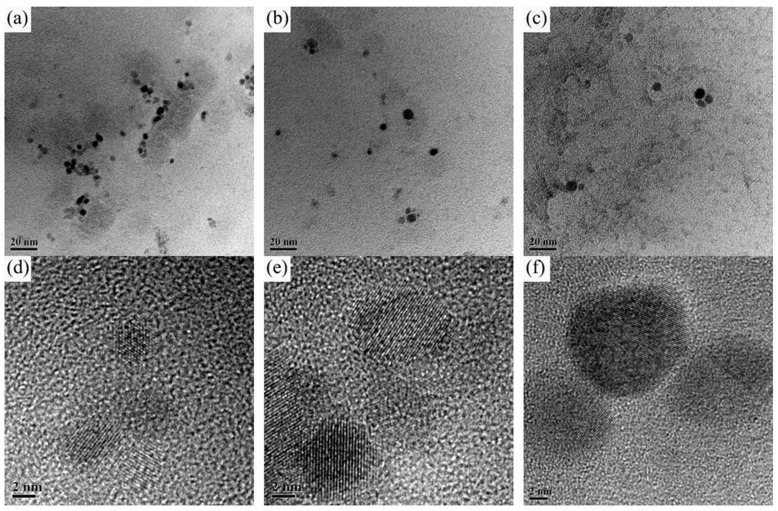
Second-phase precipitation and high-resolution diagrams of 2# steel with 0.071% V in fine-grained HAZ of 950 °C under different heat inputs: (**a**,**d**) 10 KJ/cm; (**b**,**e**) 25 KJ/cm; (**c**,**f**) 35 KJ/cm.

Figure 13 displays the second-phase precipitation and the corresponding high-resolution diagrams of 2# steel with 0.071% V in fine-grained HAZ of 950 °C under different heat inputs. It can be seen that as the heat input increased, the number of nanoscale precipitates with a size of less than 10 nm decreased, while the particle size increased. Thus, the precipitation strengthening function by the nanoscale second phase was reduced, resulting in a decrease in microhardness in fine-grained HAZ. 

On the one hand, as the heat input increased, the cooling time of the thermal cycle increased, and the residence time at high temperature increased, so that part of the second-phase particles was coarsened. On the other hand, the cooling rate decreased, and the driving force of precipitation decreased, which led to a reduction in the amount of precipitation. Therefore, the increase in welding heat input led to the weakening of the precipitation strengthening by the second phase, which was harmful in reducing the softening of the fine-grained HAZ.

In summary, for deep-sea pipeline steel with V microalloying, increasing the V content and reducing the welding heat input could reduce the effective grain size of fine-grained HAZ, and increase both the proportion of low-angle grain boundary and dislocation density. At the same time, the particle size of the nanoscale second phase precipitated in microstructure was reduced, while the number was increased. As a result, the microhardness of the fine-grain HAZ was significantly increased, and the HAZ softening problem was effectively resolved. 

## 4. Conclusions

(1) There was a softening zone in the fine-grained heat-affected zone (HAZ) of 900–1000 °C for the deep-sea pipeline steel by industrial production.
(2) In this work, adding 0.025% V effectively reduced the softening problem of fine-grained HAZ. The effect was obvious when the V content was increased to 0.071%.
(3) The improvement of the microhardness in fine-grained HAZ through V microalloying was attributed to the following reasons: (1) the microstructure of fine-grained HAZ was refined, while both the substructure and dislocation density were increased; and (2) the nanoscale precipitation of the second phase was promoted, resulting in significant precipitation strengthening.
(4) Using a low welding heat input of 10KJ/cm could effectively reduce the softening phenomenon of the fine-grained HAZ compared with heat inputs of 25 and 35 KJ/cm.

## Figures and Tables

**Table 1:** Chemical composition of experimental steel (wt. %).

No.	C	Mn	Si	P	S	Nb	V	Ti	Mo	Ni	Cr	Cu
0#	0.045	1.71	0.21	0.007	0.0014	0.059	/	0.0015	0.17	0.22	0.20	0.14
1#	0.049	1.76	0.19	0.0053	0.0041	0.052	0.025	0.0011	0.25	0.20	0.21	0.21
2#	0.053	1.77	0.19	0.0058	0.0041	0.054	0.071	0.0097	0.25	0.21	0.21	0.21

**Table 2:** Mechanical properties of experimental steel (wt. %).

No.	R_t0.5_ (MPa)	R_m_ (MPa)	R_t0.5_/R_m_	A (%)
0#	481	628	0.76	33
1#	497	630	0.79	30
2#	505	636	0.79	28

## Data Availability

We confirm that the data supporting the findings of this study are available within the article.
